# Alcohol Hangover Has Detrimental Impact Upon Both Executive Function and Prospective Memory

**DOI:** 10.3389/fpsyt.2019.00282

**Published:** 2019-05-17

**Authors:** Thomas Heffernan, Abby Samuels, Colin Hamilton, Michael McGrath-Brookes

**Affiliations:** ^1^Department of Psychology, Northumbria University, Newcastle upon Tyne, United Kingdom; ^2^School of Health and Social Care, London South Bank University, London, United Kingdom

**Keywords:** alcohol hangover, executive function, prospective memory, young adults, memory function

## Abstract

The alcohol hangover (AH) is a state of general malaise following an evening of heavy episodic drinking when the blood alcohol concentration of the person reaches/approaches zero. The aim of the current study was to investigate what impact the AH has upon both executive function (EF) and prospective memory (PM). Previous research has shown that the AH has a detrimental effect upon cognitive abilities, including attention, working memory, and PM. The current study focused upon what impact AH might have upon both EF and related PM in the same cohort, both of which underpin everyday remembering. The current study compared an AH group (AHG) with a non-hangover group (NHG) on both EF and PM measures. Forty-one participants aged 18–29 years were tested; 19 comprised the AHG and 22 of whom made up the NHG (individuals who reported no heavy drinking the day before and did not report any significant hangover symptoms). A Verbal Fluency task measured EF and the Prospective Remembering Video Procedure measured PM. The Acute Hangover Rating Scale measured AH symptoms and severity, and a Digital Breath Analyzer Test measured their blood alcohol concentration (BAC). A Recreational Drug Use Questionnaire measured alcohol and other drug use. Anyone reporting having used an illicit substance across their lifetime (e.g., cannabis, ecstasy) or who smoked heavily were omitted from the study. Two univariate analyses of covariance compared the AHG and NHG groups on Verbal Fluency and Prospective Remembering Video Task scores (controlling for age, total alcohol units consumed per week, and the number of years spent drinking). The AHG recalled significantly fewer items on the Verbal Fluency task [*F*_(1, 36)_ = 7.42, *p* < 0.01] and on the Prospective Remembering Video Task NHG [*F*_(1, 36)_ = 14.9, *p* < 0.001] when compared with the NHG. Overall, it appeared that a state of AH significantly impaired both EF and PM. Given the importance of EF and PM to everyday remembering, these findings may have farther-reaching implications.

## Introduction

The alcohol hangover (AH) is a condition characterized by a combination of symptoms including physical symptoms, such as headache, drowsiness, tremulousness, nausea, dry mouth, gastro-intestinal complaints, fatigue; and mental symptoms, such as hyper-excitability, anxiety, poor concentration, and cognitive deficits (1–2). This general feeling of malaise is the result of a bout of heavy episodic drinking (typically drinking in excess of four alcoholic drinks for women and five drinks for men), the symptoms of which are experienced 6–8 h after the bout of heavy drinking, starting when the blood alcohol concentration of the person reaches or approaches zero (3). Cognitive deficits are among the most prominent symptoms accompanying a state of AH (2). Although findings from early research on the impact of the AH upon cognition have been impeded by many methodological limitations, including the introduction of expectancy effects and the lack of adequate (e.g., non-hangover) comparison groups (4–6), more recent work has found consistent findings from both laboratory-based studies and naturalistic studies of the AH. AH-related impairments have been found in memory, attention, and psychomotor performance, compared with non-hangover controls (7–11). For example, recent research has observed AH-related performance deficits in sustained attention and attentional selection (12–13), slower choice reaction times (14), reduced short-term recall for both digit and visual patterns (15), as well as impairments in spatial and numeric working memory and inhibition (16). It is clear that the majority of this previous research on AH-related cognitive deficits has tended to focus on retrospective memory function—which refers to the learning, consolidation, retention, and retrieval of previously presented target material, with little published research on what impact the AH might have upon everyday memory—of which *prospective memory* (PM) is a good example. PM involves planning and remembering to execute a particular behavior at some future point in time (17), for example, remembering to carry out the correct sequence of actions so that one can work effectively, or remembering everyday tasks, such as meeting with friends, or remembering to take an important medication on time. PM is therefore seen as critical to everyday remembering and living an independent life (18).

In a recent study, AH-related deficits have been found in PM performance when compared with non-AH control group. In this research (19), an AH group was compared with a non-hangover group (NHG) using the Prospective Remembering Video Procedure (PRVP). The PRVP is a laboratory-based PM task during which the participant is required to remember a series of location–action combinations, which would later feature on a 10-min CD clip of a busy shopping high street viewed by the participant. It was concluded that PM deficits are associated with a state of AH. The evidence that there are AH-related deficits in sustained attention and attentional selection (12–13), slower choice reaction times (14), as well as deficits in working memory performance and inhibition (16) all suggest that *executive function* (EF) deficits may be implicated in such performance deficits. EF refers to a set of cognitive processes that facilitate planning, attention, initiating appropriate actions, inhibiting inappropriate stimuli, as well as the manipulation of information within working memory (20–21). There is good evidence that EF and PM are intimately related. For example, performance on PM tasks relies heavily on prefrontal systems in the brain and the integrity of related EF (22–23). Frontally mediated EF is believed to play key roles in a range of processes, including planning a task, monitoring one’s environment, the inhibition of extraneous impulses, and cognitive flexibility, all of which will have an impact upon successful PM functioning (24–25). Both EF and PM play crucial roles in everyday functioning, such as healthy living and ageing (26–29), and therefore a compromised PM (due to a state of AH) should also be accompanied by deficits in related EF.

Based on the premise that EF and PM share similar cognitive resources, it was hypothesized that deficits in both sets of memory processes would be evident in an AH group when compared with a non-AH group. A semantic Verbal Fluency task was used to measure EF in the current study, which required the participant to recall as many unique words as possible within a 1-min period with no repetitions. Semantic verbal fluency is a valid measure of both verbal working memory and EF (30). PM was measured using the PRVP, which is a valid and reliable test of PM and has been used successfully in previous research to determine a variety of drug-related impairments in PM, including deficits associated with cannabis use (31), ecstasy (32), binge drinking (33), as well as a state of AH (19). It was predicted that both EF and PM deficits would be associated with a state of AH when compared with a non-hangover control group. Since other drug use (31–33) can affect PM independent of a state of AH, anyone using an illicit drug or who smoked heavily was excluded from the study. Age, total alcohol use in terms of the number of units per week consumed, and years spent drinking were included as covariates in the analyses.

## Methods and Materials

### Participants

Recruitment for the study was *via* advertisement among university students and by word of mouth. Forty-one participants were included in the study, all of which were young adults aged between 18 and 29 years. Nineteen of these made up the AH group (AHG—mean age = 22.7 years, SD = 2.50). A state of AH was defined here as someone who experienced two or more biological, physiological, and/or affective symptoms (the minimum number of symptoms used as a baseline was taken from previous research (1), such as headache, drowsiness, tremulousness, poor concentration) the day after a single episode of heavy drinking, beginning when the blood alcohol concentration reached/approached zero. Twenty-two participants constituted the NHG (mean age = 22.1 years, SD = 3.48) and were individuals who reported not having engaged in a heavy bout of drinking the night before testing and did not report any significant hangover symptoms. The allocation to the AHG or NHG was made after testing was completed. Participants were unpaid volunteers made up of students studying at a university in the northeast of England. A chi-square analysis revealed that there was no significant difference in terms of sex of participant between the AHG (7 males, 12 females) and the NHG (6 females, 16 males; χ^2^ = 0.43, df = 1, p = 0.73). Anyone who reported having used an illicit substance (e.g., cannabis, ecstasy) in the past or was using the substance currently was excluded from the study. This reduced the initial sample of 80 participants tested to the 41 included in the study. Tobacco smoking was minimal within each group, with only a handful of participants reporting smoking less than 10 cigarettes per week. Each participant also had the opportunity to report any clinical condition (e.g., clinical depression, alcohol dependence, etc.) that he/she may be suffering from or had suffered from previously. None did so.

### Design

The study adopted a between-subjects design comparing the AHG and NHG on EF and PM. There were two main dependent measures; the first was the total score on the Semantic Verbal Fluency Task and the second was the total score on the PRVP. Age, the number of alcohol units consumed per week, and the number of years spent drinking were included in the main analyses as covariates. The order of presentation of the test materials remained constant across the participants.

### Materials

#### Executive Function

A semantic verbal fluency was used to measure EF, which is a short test of verbal functioning and is seen as a valid measure of both verbal working memory and executive control (30, 33). In the current study a semantic Verbal Fluency task was used, which required the participant to recall as many words from the category “FRUIT” within a 1-min period and without any repetitions; the higher the score, the more proficient the EF.

#### Objective Prospective Memory

PM was assessed using the PRVP. The PRVP is a laboratory-based measure of PM based on a methodology developed and used by earlier researchers to study the impact of a range of drugs that impact upon PM, such as the deleterious effect of cannabis use, binge drinking, and smoking upon PM (30–32). For the PRVP task, each participant was required to read a series of 12 location–action combinations for 1 min and commit these to memory. They were told that the location–action combinations would feature in a short (10-min) CD clip of a busy shopping high street and that they were required to write down on a black response sheet as many location–action combinations that they could remember while viewing the CD clip, but only when the familiar location was reached on viewing the CD clip and not before. Examples of these location–action combinations include “When you reach a shop called the Card Store” (location), “Ask directions to the train station” (action) and “When you see a woman sat on a bench with a dog” (location), “Note the colour of the bag she was carrying” (action). The researcher monitored the participant to ensure that they followed the correct procedure. One point was scored for each successful location–action combination recalled, with a maximum total score of 12 points; the higher the score, the more proficient the PM. In order to increase the ecological validity of the task and to make it more akin to real life, the participant was instructed to engage in a secondary task in which they were asked to know how many people accompanied a pushchair or pram that appeared in the CD clip. This secondary task was not assessed or analyzed, but was merely included to provide a dual task paradigm, increasing the ecological validity of the task (18).

#### Alcohol and Other Substance Use

Alcohol and other substance use was assessed using a modified version of the University of East London Recreational Drug Use Questionnaire (referred to here as the RDUQ). The RDUQ asks the participant to record his/her alcohol use in terms of the number of units consumed per week. To aid in this, the following UK guidelines were provided: 1 unit of alcohol equates to 1–2 pints of normal strength beer, a standard 125-ml glass of wine or 1 × 25 ml measure of spirit, with each unit being equal to approximately 8 g or 10 ml of pure alcohol. The researcher was on hand to provide any further guidance required by the participant in order to calculate their alcohol use. The RDUQ was also used to measure the participant’s last alcohol use in hours and how many years they had been drinking. Similar details of other drug use (e.g., smoking, ecstasy, cannabis) were also recorded. The RDUQ has been used in previous research on alcohol and other drug use (19, 30–32). The participant also had the opportunity to list any previous or current clinical condition (e.g., depression, alcohol dependence, etc). None reported having done so.

#### Alcohol Hangover Symptoms

The Acute Hangover Scale (34) is a self-report measure of the number and severity of the AH symptoms experienced, for example, thirsty, tired, headache, dizziness, loss of appetite, and gastrointestinal problems, ranging from zero symptoms to nine symptoms reported. In the present study and based on a previous work (1), the presence of two or more of these symptoms was taken to indicate the presence/absence of a state of AH (along with a blood alcohol concentration that had reached zero/or was close to zero). The severity of these symptoms was also calculated, with severity being rated from zero (none) to seven (where the severity was deemed to be “incapacitating”), with an average severity rating calculated (across the total number of symptoms reported) for each participant.

#### Breath Analyzer

The Draeger AlcoDigital 3000 digital breath analyzer measured the alcohol content of each participant’s breath; this was used to confirm that each participant’s blood alcohol concentration had returned to zero/or was close to zero.

### Procedure

The study was approved by the Life Sciences Ethics Committee at Northumbria University and was in accordance with the British Psychological Society Code of Ethics. Written informed consent was obtained from each participant. The AHG group consisted of individuals who had reported engaging in a heavy bout of drinking the night before testing. The NHG stated that they have not been out drinking the previous night. Testing took place on an individual basis in a quiet and undisturbed laboratory setting. Each participant was presented with the PRVP task first, followed by the EF task; they were then asked to complete the acute AH scale and substance use questionnaire, and were finally asked to complete the alcohol breath test. A state of AH was determined by the presence of two or more AH symptoms and a blood-alcohol concentration of zero/approaching zero as measured approximately 8–10 h after their drinking session. The procedure took approximately 20 min to complete. Following participation, each participant was thanked for their cooperation and provided with details of how they could withdraw their data if they so wished (none did so) and how they could obtain overall findings from the study.

### Analysis

Two independent t-tests were applied to the data in order to confirm whether the AHG differed from the NHG in terms of the number of AH symptoms experienced and the severity of these symptoms. Two univariate analyses of covariance (ANCOVAs) were applied to the Verbal Fluency (EF task) and Prospective Remembering Video Task (PM task) scores (controlling for age, the number of alcohol units consumed per week, and the number of years spent drinking) in order to compare performance on the main EF and PM tasks between the AHG and NHG.

## Results

**Table 1** contains the means and standard deviations (in brackets) comparing the AHG and NHG on the number of AH symptoms and severity of these symptoms, age, the number of alcohol units consumed per week, the number of years spent drinking, and scores on the Verbal Fluency Task and Prospective Video Remembering Task (PRVP).

**Table 1:** Means (and standard deviations) comparing the AHG and NHG on the number of alcohol hangover symptoms and severity of these symptoms, age, the number of alcohol units consumed per week, the number of years spent drinking, and scores on the Verbal Fluency Task and on the Prospective Video Remembering Task (PRVP).

	AHG (n = 19)	NHG (n = 22)
Hangover symptoms Hangover severity Age	6.68 (1.15) 3.35 (3.84) 22.7 (2.50)	1.31 (0.94)* 0.44 (0.28)* 22.1 (3.48)
Alcohol units per week	19.9 (7.92)	14.2 (9.33)
Years drinking alcohol	6.73 (2.84)	5.81 (3.33)
Verbal fluency scores	12.3 (3.66)	15.2 (3.11)*
PRVP scores	4.00 (1.88)	7.18 (2.48)*

* = significant at the p < .05 level.

AHG, alcohol hangover group; NHG, non-hangover group; PRVP, Prospective Remembering Video Procedure.

For all inferential testing, a cutoff of *p* < .05 was used. An independent samples t-test revealed significantly more AH symptoms reported in the AHG compared with the NHG [*t*_(39)_ = 16.3, *p* < .05; 6.68 vs. 1.31 for AHG and NHG, respectively]. A second independent samples t-test revealed significantly greater severity scores reported in the AHG compared with the NHG [*t*_(39)_ = 3.74, *p* < .05; 3.35 vs. 0.44 for AHG and NHG, respectively]. An ANCOVA applied to the verbal fluency scores (controlling for age, the number of alcohol units consumed per week, and the number of years spent drinking) revealed significantly fewer items remembered on the Verbal Fluency (EF) task by the AHG when compared with the NHG [*F*_(1, 36)_ = 7.42, *p* < .05; 12.3 vs. 15.2 for AHG and NHG, respectively]. A second ANCOVA applied to the PRVP scores (controlling for age, the number of alcohol units consumed per week, and the number of years spent drinking) revealed significantly fewer items remembered on the PRVP (PM) task by the AHG when compared with the NHG [*F*_(1, 36)_ = 14.9, *p* < .05; 4.00 vs. 7.18 for AHG and NHG, respectively].

## Discussion

Previous research has shown that a natural state of AH has a detrimental effect upon cognitive abilities, including psychomotor performance, memory, and attentional deficits (12–16), many of which functions rely on effective EF (20–21). More recently, research has shown that the AH also impedes everyday memory in the form of PM (19). Since EF and PM are intimately related and rely on similar cognitive resources (22–25), it was hypothesized that deficits in both sets of memory processes would be associated with a natural state of AH. For this purpose, the current study compared an AHG with a NHG on an objective measure of EF in the form of a Verbal Fluency task (30) and an objective measure of PM in the form of the PRVP used in previous research (19, 30–32). The results demonstrated that being in a state of AH had a significant detrimental effect on both EF and PM performance. The findings were observed after omitting anyone who reported using an illicit substance (e.g., ecstasy, cannabis) or who smoked heavily, and after statistically controlling for variations in age, the number of units of alcohol consumed per week, and the number of years spent drinking. This is the first study to demonstrate both EF and PM deficits associated with a state of AH in the same cohort. Based on the current findings, both EF and PM deficits should be added to the growing list of neuropsychological sequelae associated with a state of AH. In addition, scores on the EF and PM tasks were significantly correlated, reinforcing the notion that both sets of cognitive processes are closely related (22–25) and rely on similar cognitive resources.

This study is important as it contributes to a limited number of studies investigating the impact a state of AH has upon cognition and memory, focusing here specifically on EF and related PM. PM has both a retrospective and prospective component (17–18). For example, remembering to perform a particular action at an appropriate time in the future (e.g., remembering to visit the shops before going home) represents the prospective element, whereas remembering the content of the action (e.g., what items to buy) represents the retrospective element. Executive processes play an important role in accessing long-term memory, but may be especially important for the prospective component of PM. Therefore, good EF and PM functioning is pivotal to proficient human functioning in terms of everyday remembering and successful independent living. Given that good EF and PM function plays an important role in all walks of life, including healthy adolescent development (35), efficient performance in the workplace (36–37), and healthy ageing (38–40), then impairments or reduced functioning as a result of a state of AH can significantly compromise both EF and PM performance within these domains.

Although the current study has highlighted both EF and PM deficits associated with a state of AH, it is not without its limitations. Biological drug-screening methods would provide a more accurate view of other drug-use habits, rather than relying on self-reported measures of drug use (41). Future research should also include a non-alcoholic group so that EF and PM performance in these two alcoholic groups could then be compared with a nondrinking control group. This would enable us to determine whether the NHG has sufficiently intact EF and PM capacities similar to that of nondrinking controls. Although the current study used valid and reliable methods for measuring both EF and PM, other measures of EF and PM should be used in order to confirm the findings from the current study, such as the Reverse Digit Span Task for measuring EF (42) and the Cambridge Prospective Memory Test for assessing PM (43). Also, the current study could be improved by including subjective accounts of cognitive impairments associated with a state of AH, which would provide a more comprehensive insight into the objective and subjective deficits associated with a state of AH. The relatively small sample size is also a limitation of the study; this was partially due to the omission of anyone who used an illegal substance (e.g., cannabis, ecstasy) across their lifetime and may have affected the power of the study. Given that heavy episodic drinking can begin as early as 12 years of age (44), future research should examine what impact a state of AH (or repeated states of AH) has upon a child’s neurocognitive development, especially since the brain is still developing across this critical period of time (45–47). Any injurious impact upon this development because of a state of AH could interfere with related cognitive processes, including EF and PM, producing long-lasting deficits to brain structure and its associated cognitive function.

There are a number of clinical implications resulting from a state of AH. For example, a state of AH commonly goes underdiagnosed in the general population and can have serious consequences in terms of physical, psychological, and occupational consequences (48). In addition, it is predictive of future risky drinking patterns and poor academic performance, as well as risky behaviors such as arrests for driving under the influence, risky sexual behavior, and sexual victimization (49). It is therefore important to understand the impact of the AH in order to guide interventions and inform patients of the consequences of the AH.

## Conclusion

The current findings demonstrate that both EF and PM deficits are associated with a state of AH. Both EF and PM deficits should be added to the growing list of cognitive sequelae associated with a state of AH. Such findings may prove useful in educating young people as to the adverse cognitive consequences of being in a state of AH and could also be used to better inform medical staff and clinicians who work with people suffering from alcohol dependence (48).

## Ethics Statement

The study was approved by the Life Sciences Ethics Committee at Northumbria University and was in accordance with the British Psychological Society Code of Ethics. Written informed consent was obtained from each participant.

## Author Contributions

TH and AS designed the observational study and conducted the required data analysis. TH, AS, and CH wrote the draft version and the final version of the manuscript. MMG contributed to the draft and final versions of the manuscript.

## Conflict of Interest Statement

The authors declare that this research received no commercial or financial support that could be construed as a potential conflict of interest.
